# Land-Use System and Forest Floor Explain Prokaryotic Metacommunity Structuring and Spatial Turnover in Amazonian Forest-to-Pasture Conversion Areas

**DOI:** 10.3389/fmicb.2021.657508

**Published:** 2021-04-21

**Authors:** Fernando Igne Rocha, Thiago Gonçalves Ribeiro, Marcelo Antoniol Fontes, Stefan Schwab, Marcia Reed Rodrigues Coelho, José Francisco Lumbreras, Paulo Emílio Ferreira da Motta, Wenceslau Geraldes Teixeira, James Cole, Ana Carolina Borsanelli, Iveraldo dos Santos Dutra, Adina Howe, Aline Pacobahyba de Oliveira, Ederson da Conceição Jesus

**Affiliations:** ^1^Department of Soil, Universidade Federal Rural do Rio de Janeiro, Seropédica, Brazil; ^2^Department of Agricultural and Biosystems Engineering, Iowa State University, Ames, IA, United States; ^3^National Agrobiology Research Center, Embrapa Agrobiologia, Seropédica, Brazil; ^4^National Soil Research Center, Embrapa Solos, Rio de Janeiro, Brazil; ^5^Department of Plant, Soil and Microbial Sciences, Michigan State University, East Lansing, MI, United States; ^6^Department of Veterinary Medicine, Universidade Federal de Goiás, Goiânia, Brazil; ^7^Department of Support, Production and Animal Health, Universidade Estadual Paulista, Araçatuba, Brazil

**Keywords:** Amazonia, tropical rainforest, 16S rRNA gene, next generation sequencing, microbial biodiversity, land-use change, prokaryotes

## Abstract

Advancing extensive cattle production is a major threat to biodiversity conservation in Amazonia. The dominant vegetation cover has a drastic impact on soil microbial communities, affecting their composition, structure, and ecological services. Herein, we explored relationships between land-use, soil types, and forest floor compartments on the prokaryotic metacommunity structuring in Western Amazonia. Soil samples were taken in sites under high anthropogenic pressure and distributed along a ±800 km gradient. Additionally, the litter and a root layer, characteristic of the forest environment, were sampled. DNA was extracted, and metacommunity composition and structure were assessed through 16S rRNA gene sequencing. Prokaryotic metacommunities in the bulk soil were strongly affected by pH, base and aluminum saturation, Ca + Mg concentration, the sum of bases, and silt percentage, due to land-use management and natural differences among the soil types. Higher alpha, beta, and gamma diversities were observed in sites with higher soil pH and fertility, such as pasture soils or fertile soils of the state of Acre. When taking litter and root layer communities into account, the beta diversity was significantly higher in the forest floor than in pasture bulk soil for all study regions. Our results show that the forest floor’s prokaryotic metacommunity performs a spatial turnover hitherto underestimated to the regional scale of diversity.

## Introduction

Habitat fragmentation and land-use changes have led to an alarming and rapid decline of biodiversity in tropical ecosystems (Nobre et al., 2016). Soil microbiomes, which are vital to ecosystem functioning and comprise a great capacity to reflect the impact of the land-use intensification on natural resources (Barnes et al., 2017), are one of the affected components of this biodiversity (Ushio et al., 2010; Aponte et al., 2013). Consequently, it is crucial to understand how the conversion of tropical forests to other land-use systems affects edaphic microbiota, especially prokaryotes (Hug et al., 2016). Previous studies have identified a strong relationship between bacterial biodiversity, soil properties, and land-use systems in the Amazon rainforest (Jesus et al., 2009; Rodrigues et al., 2013; Mendes et al., 2015; Navarrete et al., 2015; de Carvalho et al., 2016; Pedrinho et al., 2019). These findings have shown that deforestation followed by the introduction of pastures and agricultural systems increase the alpha “local” diversity (average sample diversity) of soil bacteria, contrary to the previous expectation that bacterial diversity would be positively correlated with plant diversity (Prober et al., 2015). Moreover, these studies have shown that the consequent increase in soil pH by the land-use conversion is one of the main abiotic factors shifting microbial community structure and diversity.

A still unresolved question is whether intensification of converted tropical ecosystems may contribute to soil microbial homogenization across space (Petersen et al., 2019), declining the beta diversity (average dissimilarity in composition among sub-communities) (Anderson et al., 2006). Available studies suggest that, although land-use intensification tends to increase microbial alpha diversity, this effect does not persist on the beta diversity scale, possibly decreasing the gamma “regional” diversity (total observed diversity of all samples within a given land-use) (Walters and Martiny, 2020), and a decline in microbial turnover across space (Rodrigues et al., 2013; Mendes et al., 2015; Goss-Souza et al., 2017). However, contrasting results (Lee-Cruz et al., 2013; de Carvalho et al., 2016) indicate higher components of diversity (alpha, beta, and gamma) over more intensive land uses due to the increased environmental heterogeneity, evidencing contrary trends to microbial homogenization after the land-use change.

Previous studies have been carried out with a low variety of soil types, which reduces the ability to predict different drivers in the structuring of microbial communities, besides being predominantly limited to the topsoil (i.e., borderline range between soil profile and its top organic layers). Nonetheless, organic horizons are known to sustain ecosystem functioning, especially in tropical forests (Sayer and Tanner, 2010) that predominantly grow on low-fertility soils (Grubb, 1995). Some recent efforts have investigated how microbial communities in the litter interact with the soil microbiota (Buscardo et al., 2018; Ritter et al., 2018, 2020), but it is still unknown how microbial communities in the tropical forest floor (association between litter, root layer, and bulk soil) respond to regional scales of diversity. Moreover, clearing techniques traditionally used to remove the forest involve burning most of its biomass and are the principal deforestation method in Amazonia (Brando et al., 2020). Thus, filling this knowledge gap is essential to measure the effects of biodiversity loss in tropical rainforests.

In this study, we tackled how prokaryotic metacommunity (i.e., microbiota assemblies from spatially different sites) in the Western Amazonian forest floor contributes to spatial turnover and gamma “regional” diversity. We hypothesize that the lower alpha microbial diversity of the forest soil, reported in previous studies, is a sampling artifact caused by the non-inclusion of the forest floor as a whole, that is, by not taking into account its organic layers. We also hypothesized that the beta and gamma diversities are higher in the forest floor’s prokaryotic community than in the pasture bulk soil. We took advantage of a broad Amazonian pedodiversity, ranging from a patch of natural nutrient-rich soils in the state of Acre (e.g., Luvisols) to those with a high weathering degree in the state of Amazonas (e.g., Acrisols and Ferralsols) to test whether soil type rather than land-use history is a significant factor structuring prokaryotic metacommunity. To investigate these effects, we targeted the 16S rRNA gene using amplicon/barcode sequencing to assess microbiomes in a geographic gradient that covers an extensive range of soils and landscapes in the Western Amazonia under the effects of recent forest-to-pasture conversion.

## Materials and Methods

### Sampling and Experimental Design

This study was carried out in the Brazilian Western Amazonia, within a geographical range of ±800 km, which covers spots near the cities of Bujari (state of Acre, 9°49′22″S, 67°56′51″W, elevation 196 m), Boca do Acre (state of Amazonas, 8°44′26″S, 67°23′3″W, elevation 99 m) and Manicoré (state of Amazonas, 5°48′34″S, 61°18′2″W, elevation 32 m) (Supplementary Figure 1). The climate of the region, characterized by tropical monsoon rain and a brief dry period between June and August, is classified as “Am” according to the Köppen system. The annual average rainfall varies between 2200 and 2800 mm, and the average annual temperature varies between 24 and 26°C (Alvares et al., 2013). The parent materials for soils in the Western Amazon region are mixed-textured Tertiary and Quaternary fluvial sediments of Andean origin (Rodrigues, 1996). The sites were selected based on their importance for tropical forest conservation and the rapid advance of livestock production, which has been reported as one of the main drivers of deforestation. Sampling took place in August 2017 following the Sustainable Amazonia Network’s experimental design (Gardner et al., 2013), with a total of 65 sampling points distributed among five forests and eight pasture areas (Supplementary Table 1). We used 200 m linear transects, including five sampling points equally spaced 50 m apart. Composite soil samples were collected at each sampling point for both molecular analysis and soil characterization. Three pooled subsamples formed each composite sample.

**FIGURE 1:**
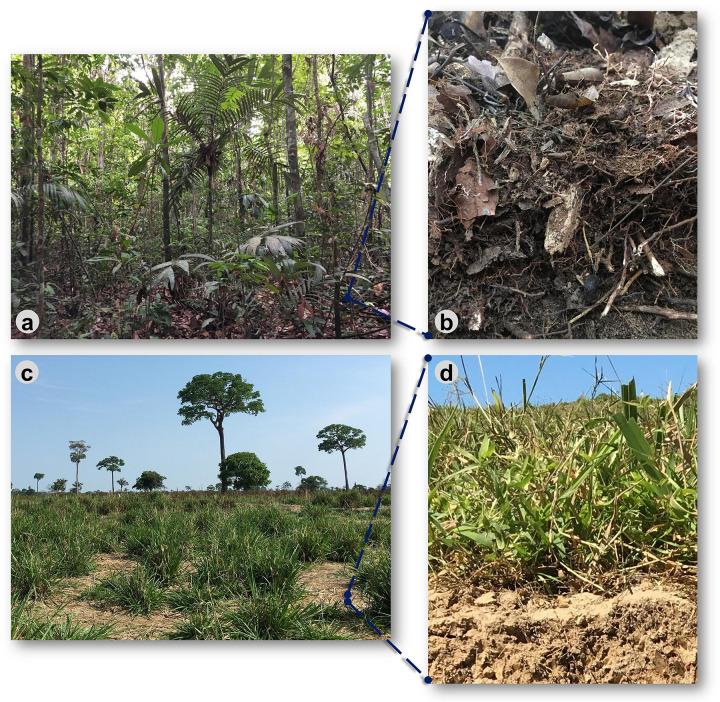
Illustrative representation of the evaluated land uses in the Brazilian Western Amazonia, focusing on the forest floor’s decay after converting the forest to pasture. **(a)** Rainforest where the **(b)** forest floor (litter and the root layer on top of the mineral bulk soil) were sampled; **(c)** pasture systems, and **(d)** their respective soil surface with a reduced presence of organic layers.

Traditional sampling for molecular microbial ecology studies usually removes the litter before sampling (Mendes et al., 2015; de Carvalho et al., 2016; Khan et al., 2019; Pedrinho et al., 2019). Nevertheless, when visiting our study sites, we observed that the forest floor has a root layer on top of the mineral soil core, which is intertwined with particulate organic matter and decomposed litter. This layer is thicker and has similar aspects to an H horizon (Figure 1) in some forests, such as in Manicoré/AM. For this reason, we stratified samples in the forest floor into litter (leaves, mostly), root layer, and the mineral bulk soil (soil A-horizon at a depth of 0–10 cm; hereafter bulk soil). Sampling was done at each sampling point of the linear transect, also formed by three pooled subsamples. The forest root layer was involved by particulate organic matter, which was recovered by sieving (2 mm mesh) and used for DNA extraction. Only the bulk soil (0–10 cm) was sampled in pasture lands since no superficial root layer nor a significant litter component existed in these systems. All material sampled for molecular analysis was immediately packed in sterile pouches and refrigerated at −80°C in the shortest time possible.

### Chemical and Physical Analysis

Soil classification was performed for all evaluated sites, using one profile per transect where pedological description and horizon soil sampling were carried out (Santos et al., 2005; WRB, 2015; dos Santos et al., 2018). Soil physical attributes (particle size distribution and flocculation degree) were determined by the sedimentation method and reading by densimeter from the sample dispersion with 0.1 mol L^–1^ sodium hydroxide solution. The chemical analyses consisted of pH in water and KCl 1 mol L^–1^, determined potentiometrically, in the soil: 1:2.5 solution with 1 h of contact and agitation of the suspension at the time of reading. Exchangeable sodium and potassium (Na^+^ and K^+^) were extracted with HCl 0.5 mol L^–1^ + H_2_SO_4_ 0.0125 mol L^–1^ (Mehlich^–1^), in the proportion of 1:10 and determined by photometry of flame emission. The measurement of exchangeable calcium and magnesium (Ca^2+^ and Mg^2+^) was performed by atomic absorption spectroscopy and exchangeable aluminum (Al^3+^) by titration after extraction with KCl 1 mol L^–1^ in the proportion of 1:10. The determination of potential acidity (H + Al) was carried out by titration after extraction with calcium acetate 0.5 mol L^–1^ in the proportion 1:10 and pH 7.0. The organic carbon was determined by titration of the remaining potassium dichromate with ammoniacal ferrous sulfate after the oxidation process. The calculation of derived correlations, i.e., total exchangeable bases (sum of bases = Ca^2+^ + Mg^2+^ + K^+^), base saturation index [BS% = 100 × S/total cation exchange capacity (CEC)], and aluminum saturation index {Al saturation = [mmolc (Al^3+^) dm^–3^ × 100]/[mmolc (effective CEC) dm^–3^]}, were also analyzed (Teixeira et al., 2017) at the National Soil Research Center, Brazil. The litter was properly ground and homogenized to quantify the N and C contents using CHN elemental analysis, besides the extraction of polyphenols and tannin content, following the Tropical Soil Biology and Fertility protocol (Anderson and Ingram, 1993) and conducted at the National Agrobiology Research Center, Brazil.

### DNA Extraction and High-Throughput Sequencing

DNA extraction from the litter, root layer, and bulk soil was performed using the standard DNeasy PowerSoil kit protocol (MO BIO Laboratories, Inc.), with adjustments in the time and beating intensity of the initial protocol step after adding material to the tubes containing the beads and solution C1 (FastPrep FP120-Thermo Savant BIO101; time = 40 s; beating = 4×). Litter and the fragmented material involving root layer samples (previously sieved in a 2 mm mesh) were macerated in liquid N with pre-sterilized mortar and pestle and maintained for a minute in a water bath. Amplification of the 16S rRNA gene for DNA samples of litter, root layer, and bulk soil was performed using barcoding DNA (Caporaso et al., 2012) with specific modifications to primer degeneracy 515F as described in Parada et al. (2016). PCR products were purified and subjected to library preparation and sequencing with Illumina MiSeq technology following the Earth Microbiome Project protocol for 16S Illumina Amplicon at the Argonne National Lab Core Sequencing Facility, United States.

### Sequencing Data Processing

Sequence separation was performed in a Python environment based on primer barcodes. The 16S rRNA sequence data were further processed, aligned, and categorized using the DADA2 microbiome pipeline^1^ by recommended parameters with quality filtering of sequence length over 250 base pairs (Callahan et al., 2016). DADA2 characterizes microbial communities by identifying the unique amplicon sequence variants (ASVs) among the 16S rRNA reads. ASVs exhibit fewer false-positive taxa and reveal cryptic diversity, otherwise undetected by traditional OTU approaches (Callahan et al., 2017). Further, the taxonomy was assigned for each ASV assessing the Silva taxonomic training (database v132) (Quast et al., 2012). R packages “dada2” v.1.14.0 (Callahan et al., 2016) and “decipher” v.2.14 (Wright et al., 2012) were used in the R 3.6.1 environment (R Team, 2018).

### Prokaryotic Metacommunity Analysis and Environmental Variable Selection

The quality step (filtering, denoising, and the removal of chimeras) on the abundance matrices was used to eliminate low prevalence sequences and sequences from Chloroplast, Eukaryota, and Mitochondria. After that, 2,735 ASVs were removed, resulting in 1,901,440 read counts, divided into 15,335 ASVs with 15,221 average counts per sample. Abundances were standardized by the median sequence depth (15,212 paired-reads). For soil variable selection, principal component analysis (PCA) was applied on the correlation matrix to obtain a smaller subset of soil variables based on their component loadings, using “factoextra” v.1.0.7 R package (Kassambara and Mundt, 2018). Non-metric multidimensional scaling (NMDS) was performed to visualize similarities among communities by factors (sites, land-use, and soil variables). The ecological distance was calculated with the Bray–Curtis dissimilarity matrix. Subsequently, the factors were compared through permutational analysis of variance (PERMANOVA) using Hellinger transformed data (Legendre and Gallagher, 2001), both with 10,000 permutations. A generalized additive model with an extra penalty (γ = 1.4) was fitted to explain each selected variable’s importance on the abundance matrix, with maximum likelihood as a smoothing parameter estimation method (Marra and Wood, 2011). The distance matrix of biotic (ASVs) and abiotic (environmental variables) data were matched using Procrustes analysis (Peres-Neto and Jackson, 2001) to measure their correlation. We used differential heat-tree to visualize significant differences in taxonomic composition between the forest floor compartments in a pairwise Wilcoxon rank-sum test comparison using the “metacoder” v.0.3.3 R package (Foster et al., 2017). Analyses were carried out in R environment, mainly supported by “phyloseq” v.1.30.0 (McMurdie and Holmes, 2013), “vegan” v.2.5-6 (Oksanen et al., 2016), and “ampvis2” v.2.5.5 (Andersen et al., 2018) packages and dependencies. Finally, linear discriminant analysis (LDA) effect size (LEfSe) (Segata et al., 2011) was accessed on MicrobiomeAnalyst (Chong et al., 2020) to incorporate statistical significance with biological consistency (effect size) estimation in a non-parametric factorial Kruskal–Wallis sum-rank test to identify features with significant differential abundance. Features with at least 2.0 log-fold changes and α < 0.05 were considered significant. All *p*-values were corrected by the false discovery rate method (Benjamini and Hochberg, 1995) to avoid the inflation of Type-I error due to multiple tests.

### Diversity Partitioning (α, β, and γ)

HCDT entropy has been proven as a powerful tool for measuring diversity by generalizing classical indices (Marcon et al., 2014). Here, it was turned into Hill numbers, which generate effective numbers of equally frequent species for each value of “q” in a unified framework, making possible the straightforward interpretation and comparison (Chao et al., 2014). The order of diversity “q” attaches different sensitivity to rare species, being: “q = 0” the most sensitive (species richness); “q = 1” all individuals are equally weighted (exponential of Shannon’s entropy); and “q = 2” is sensitive to the dominant species (inverse of Simpson index) (Jost, 2006). Because Hill numbers are continuous and have a common unit, they can be portrayed on a single graph as a function of “q,” leading to a “diversity profile” of effective species. Further details can be found in Chao et al. (2014). Diversity partitioning means that, in a given area, the gamma diversity of all individuals found can be divided internally, within the plot unit (alpha diversity) and between the local assembly (beta diversity) (Daly et al., 2018) and was calculated for all compartments of the forest floor and pasture bulk soil. Kruskal–Wallis test was used in univariate comparisons based on the global effective numbers (i.e., Hill’s q 0, 1, and 2) as a single way to highlight the contribution of each compartment and all the forest floor at a given diversity scale (alpha, beta, and gamma). Analyses were performed using the “entropart” v.1.6-1 R package (Marcon and Hérault, 2015) and “stats” v.3.6.1 (R statistical functions).

## Results

### Gradient of Soil Fertility Drives Soil Prokaryotic Metacommunity Structuring

A PCA on the selected soil variables (i.e., pH, BS%, Al saturation, Ca + Mg, sum of bases, and silt; Supplementary Figure 2) revealed 83 and 11.2% of the explained variance on PC1 and PC2, respectively. For the extracted soil variables, no statistical differences were found between the forest and pasture of BUJ and between the pastures of BAC and MAN (Supplementary Table 2).

**FIGURE 2:**
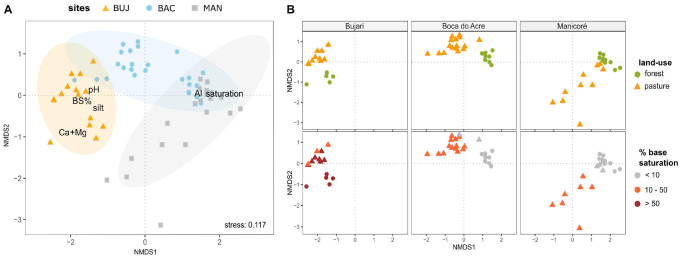
Land-use and soil type shape prokaryotic metacommunity structure in the bulk soils. **(A)** Non-metric multidimensional scaling (NMDS) based on Bray–Curtis dissimilarity among samples in the normalized ASV data of soil prokaryotic communities, highlighting the study sites and soil variables correlated with community structure; **(B)** NMDS (Bray–Curtis dissimilarity) of prokaryotic soil communities of each study area, highlighting the sample distribution pattern by land-use (upper boxes) and gradient of fertility (below boxes, by the base saturation index).

The structure of prokaryotic metacommunity in the bulk soil (i.e., microbiota assemblies from spatially different sites) differed among the study sites (PERMANOVA, *F* = 8.20, *p* < 0.001) as well as between land uses (*F* = 11.07, *p* < 0.001) for all pairwise comparisons (Supplementary Table 3). Metacommunity structure was significantly correlated to the base saturation index, showing that it shifted along a gradient of soil fertility (Figure 2; *F* = 9.93, *p* < 0.001), from places with highly weathered soils (BAC and MAN forests) to those with high natural fertility (BUJ forest and pasture). We detected a significant statistical interaction between sites and land-use (*F* = 3.97, *p* < 0.001; Supplementary Table 3), which indicates that both factors contribute to prokaryotic community structuring, influenced by the soil type by each site and land-use characteristics, as shown further.

The Procrustes analysis identified a positive correlation between biotic and abiotic matrices (71.83%, *p* < 0.001). Generalized additive models for each extracted soil variable in the PCA revealed high deviance explained for those variables, determining its importance in mediating prokaryotic communities’ distribution (Supplementary Table 4). Moreover, soil pH was positively associated with ASV richness (Supplementary Figure 3).

### Land-Use and Soil Type Shape the Predominant Composition Among Prokaryotic Soil Communities

Features that most likely explain differences between land-use systems and sites were determined by LDA LEfSe, and patterns were detected showing taxa associated with land-use regardless of soil type. At the phylum level, *Proteobacteria, Gemmatimonadetes, Thaumarchaeota, Rokubacteria*, and *WPS-2* were revealed as the most abundant in forest systems (Supplementary Figure 4). In contrast, *Actinobacteria, Chloroflexi, Firmicutes*, and *Bacteroidetes* were the phyla with the highest differential abundance in pasture systems. For BAC, we found eight significantly more abundant phyla in pasture soils and four in the forest’s bulk soil. Both BUJ and MAN had the same number of predominant phyla among their land uses. When comparing the same land-use among different sites, we observed that the BUJ forest hosts the largest significant number of predominant phyla compared to other sites. *Verrucomicrobia*, in BUJ, and *Acidobacteria*, in BAC, are the most prevalent phyla in pasture and forest soils, respectively (Supplementary Figure 5).

### The Structure and Composition of Prokaryotic Metacommunity in the Forest Floor Reflect Land-Use as a Biotic Selector

**FIGURE 3:**
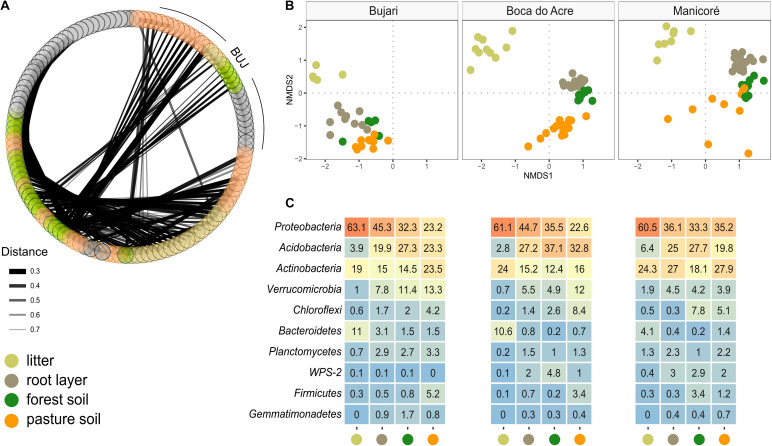
Co-occurrence of prokaryotic metacommunity between sites based on forest floor compartments and pasture bulk soil. **(A)** Co-occurrence based on compartments (litter, root layer, forest, and pasture soil) and study sites; the thickness of the links is proportional to the strength of the interactions; **(B)** NMDS by compartments and sites (BUJ, BAC, and MAN); distance measured by Bray–Curtis based on the abundance of ASVs from each sample point; **(C)** relative abundance of Bacteria (phylum level) in compartments of forest floors and pasture soil.

**FIGURE 4:**
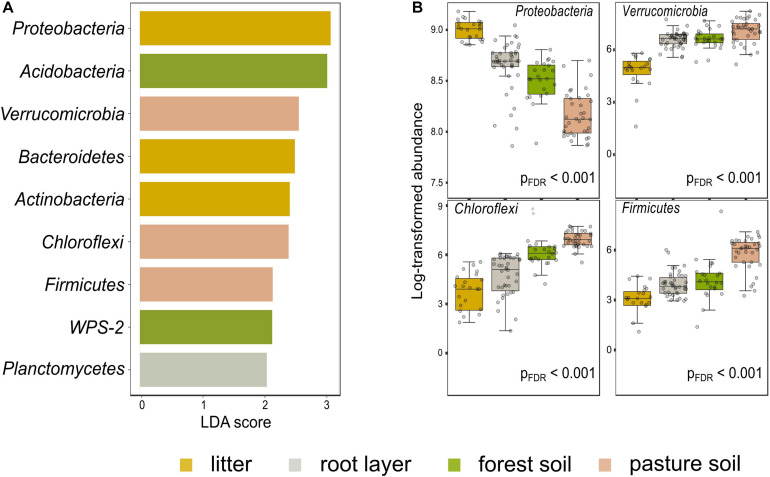
Differential abundance among the most relevant taxa in the forest floor and pasture bulk soil in the Western Brazilian Amazonia. LEfSe multivariate analysis to significant differential abundances [false discovery rate adjusted *p*-value (p_FDR_) < 0.001] with LDA > 2.0; **(A)** features selected between the compartments of the forest floor and the pasture bulk soil; **(B)** first four features based on p_FDR_ < 0.001, without the application of the LDA.

**FIGURE 5:**
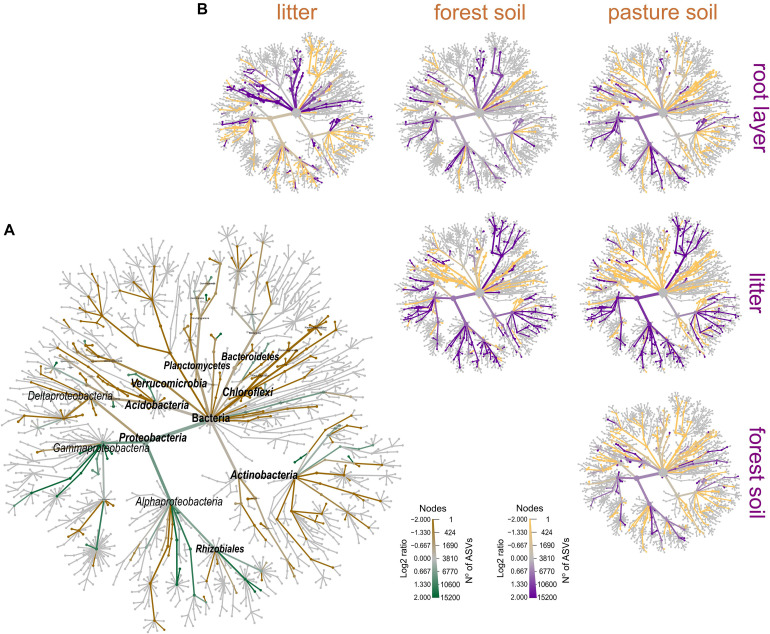
Phylogenetic differential heat tree highlighting the most expressive features among the compartments of the forest floor. **(A)** Predominance of phylogenetic groups in the forest floor (green color) and pasture bulk soil (brown color); **(B)** pairwise comparison between each compartment; the color of each branch represents the log-10 ratio of median proportions of reads observed at each compartment. Only significant differences are colored, determined using a Wilcox rank-sum test followed by a Benjamini–Hochberg (FDR) correction for multiple comparisons.

Prokaryotic metacommunity structure differed significantly among the litter, root layer, and bulk soils, and this result was consistent among all studied sites (Figure 3; PERMANOVA, *F* = 18.08, *p* < 0.001). The prokaryotic metacommunity structure of the litter communities contrasted with those found in other compartments of the forest floor (Supplementary Table 5). Differences in the prokaryotic metacommunity among sites were associated with variations in litter chemical composition (Procrustes analysis: 63.2%, *p* < 0.001), mainly due to the polyphenol content, N content, and C:N ratio (Supplementary Figure 6). All forest floor compartments were compared among themselves and with the pasture bulk soil. Taxa that were enriched or reduced were identified (Figures 4, 5). *Chloroflexi, Proteobacteria, Firmicutes*, and *Verrucomicrobia* were the most statistically different (LDA; *p* < 0.001). *Proteobacteria* was the only phylum present in all forest compartments, especially in the litter (>60% relative abundance; *p* < 0.001, LDA = 3.6). These patterns were found to be similar in all sites. *Planctomycetes* were the most representative group in the root layer of the forest (*p* < 0.001, LDA = 2.05) despite their low relative abundance (Figure 3C). Overall, 30.2% of ASVs are shared among the forest floor’s compartments; 22.6% between BAC and MAN; 13.1% between BAC and BUJ, and only 1.3% between BUJ and MAN. BUJ has 1491 (14.2%) restrict ASVs in its microbial communities (Supplementary Figure 7), reflecting the distinct chemical composition in the forest floor’s compartments in relation to the other sites.

### Forest Floor Reveals Prokaryotic Diversity and Spatial Turnover in Brazilian Western Amazonia

**FIGURE 6:**
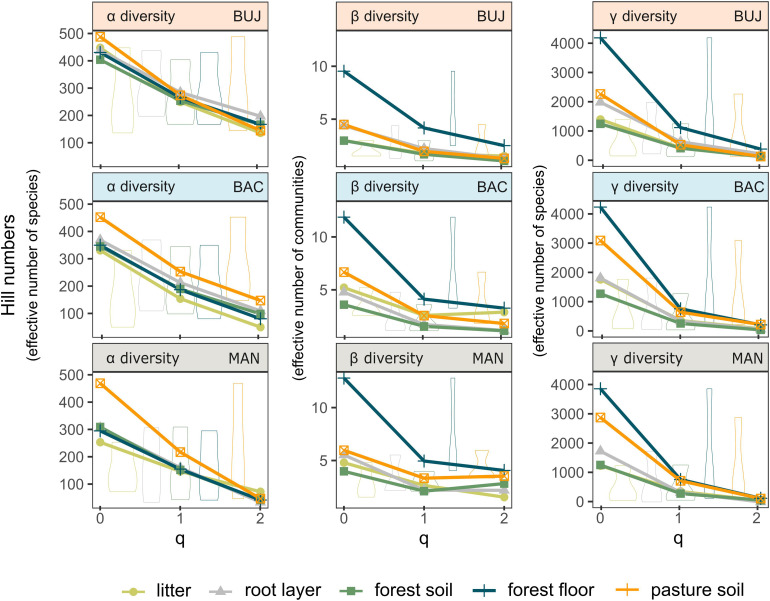
Diversity partitioning analysis evidencing the heterogeneity of prokaryotic metacommunity across land uses. Alpha (α), beta (β), and gamma (γ) (i.e., local, community, and regional) diversities for the forest and pasture bulk soils, litter, root layer, and the forest floor in each site. Hill numbers (*q* = 0, ASV richness), (*q* = 1, exponential of Shannon’s entropy for equally weighted ASVs), and (*q* = 2, inverse of Simpson index for dominant taxa).

Diversity partitioning analysis showed that the ASV richness (Hill’s q = 0) in bulk soils is significantly higher in pastures than forests for all diversity scales and study sites, especially for MAN (Figure 6). Beta (χ^2^ = 6.94, *p* < 0.001), and gamma diversity (χ^2^ = 5.43, *p* = 0.013) was also significantly higher in pasture bulk soil, except for BUJ (*p* > 0.05). The effective number of dominant ASVs was similar (Hill’s q = 2) for any diversity scale, as well as in the comparison between forests and pastures, meaning that both systems have a similar number of dominant groups in the bulk soil. Nevertheless, when the forest floor was taken as a whole, that is, when the metacommunities in the litter, root layer, and bulk soil were analyzed together, we observed that the differences in the alpha “local” diversity between forest and pastures were no longer observed, as previously found in the comparison between bulk soils (Figure 6 and Supplementary Table 7). Only BAC showed a statistically higher effective number of species in its pastures for all orders of diversity “q.” BUJ had the highest alpha diversity for both litter, root layer, and bulk soils compared to the other study sites. Especially, the ASV richness (q = 0), as well as Shannon diversity (q = 1) and Simpson dominance (q = 2) of the forest floor showed the highest beta diversity for all study sites, which indicate a more prominent spatial turnover of the prokaryotic community. For the gamma “regional” diversity, only the forest floor of BUJ had a significant global difference in the effective number of species between forest and pasture (χ^2^ = 6.64, *p* = 0.009), although the similar higher ASV richness (q = 0) in the forest floor than in the pasture bulk soil for all study regions.

## Discussion

### Prokaryotic Metacommunity Reflects the Synergistic Interaction Between Land-Use and Soil Type

Multiple analyses based on the next-generation sequencing approach allowed us to support our first hypothesis that soil type, rather than land-use patterns, mainly leads to structuring the prokaryotic metacommunity in bulk soils. This finding highlights that soil variables, especially those related to soil fertility, such as pH and base saturation, are the major attributes driving the prokaryotic community structuring in bulk soils.

Our argument is based on the observation that communities from the most distant geographic areas (±650 km; BAC to MAN) showed more remarkable structural and compositional similarities (Bray–Curtis distance = 0.51) than communities from nearby sites (±150 km; BUJ to BAC; Bray–Curtis = 0.87). This distinction reflects the influence of different soil-forming processes on microbial community structuring. Soils of the state of Acre mostly come from weathering sedimentary rocks, and specifically, those found in this study are a patch of naturally eutrophic soil, such as Luvisols (Bernini et al., 2013). Predominantly, BAC and MAN have Acrisols and Ferralsols, highly weathered soils, covering most of the Amazon basin (Schaefer et al., 2017), and developed on sandstones and claystones, and mainly formed on remnants of ferrallitic plateaus and convex hills which are not flooded (Souza et al., 2018). The gradient of soil fertility across soils with distinct pedogenesis and weathering degrees is a major contribution of this study to understanding how microbiomes are modeled under the same land-use system. Soil pH may not directly alter prokaryotic community structure but may be considered an integrating variable that provides an index of soil conditions (Lauber et al., 2009). Many soil attributes, such as nutrient availability, cationic metal solubility, organic C characteristics, soil moisture condition, and salinity, are often directly or indirectly related to soil pH (Bissett et al., 2011; Suleiman et al., 2013). However, recent studies indicate that bacterial community assembly processes differ concerning soil pH, with near-neutral pH leading to more stochastic communities, whereas extreme conditions lead to more deterministic assembly and clustered communities (Tripathi et al., 2018). Thus, the influence of variables such as temperature is mainly revealed where soil pH is relatively constant (Nottingham et al., 2018).

Our results consistently support a cause-effect relationship between soil pH and alterations in the natural structure and composition of the soil microbiomes due to the land-use conversion (Jesus et al., 2009; Mendes et al., 2015; Navarrete et al., 2015; Goss-Souza et al., 2017; Berkelmann et al., 2018). Moreover, regarding the taxonomic approach of communities, we observe a clear community fingerprint throughout land uses, even considering the different soil types. *Actinobacteria* were dominant in the pastures to the detriment of *Proteobacteria*, which were considerably abundant in the forest floor, especially in the litter. Increases in the relative abundance of *Actinobacteria* and *Chloroflexi* populations were highlighted in Fierer et al. (2012) and Mendes et al. (2015). *Actinobacteria* are functionally related to organic substrate decomposers and produce spores, allowing this group to maintain its activity in more anthropized systems (Ventura et al., 2007). Some groups of the *Chloroflexi* are thermophilic aerobes, having the ability to develop their metabolism at high temperatures, also keeping an important relationship in the decomposition of organic matter (Yamada et al., 2005) and, consequently, predominance in pasture soils. In turn, *Proteobacteria* are usually related to high levels of organic C and have been extensively reported as a land-use change indicator as its high abundance is drastically reduced after the conversion of the rainforest into pastures (Mendes et al., 2015; Navarrete et al., 2015; de Carvalho et al., 2016). *Proteobacteria*, specifically *Alphaproteobacteria*, and *Gammaproteobacteria*, which were highly evident in our study, mainly in the litter layer (see Figure 5), are functionally important in natural systems known to undergo weak soil perturbation and provide copiotroph habitats rich in recalcitrant organic matter (Pascault et al., 2013). They are also closely related to methane oxidation (CH_4_) due to their methanotrophic characteristics, helping to mitigate these gas’ emissions by controlling the production-consumption balance within systems with lower anthropic disturbance, such as forests (Tate, 2015).

### Role of the Prokaryotic Metacommunity in the Forest Floor and Deforestation as a Risk for Its Maintenance

The tropical forest floor undoubtedly plays a vital role in the biodiversity and ecosystem functioning on a global scale (Poorter et al., 2015). The biogeochemical cycles in that ecosystem regulate the most extensive terrestrial C storage, maintaining high biomass and productivity, although mainly growing on low-fertility soils (Finzi et al., 2011; Sayer et al., 2020). However, the rapid advancement of livestock expansion represents a high risk for its maintenance because the forest floor is irreversibly affected during the forest-to-pasture conversion, with no subsequent replacement of some of its compartments. Some efforts to evidence nutrient retention and uptake in the forest floor have been made (Sayer and Tanner, 2010; Sayer et al., 2020), considering that the mineral soil measurements only represent a small part of the picture. Hence, a better understanding of the role of the forest floor’s prokaryotic communities and how they are impacted by deforestation is essential to predict consequences in the face of global changes (Lladó et al., 2017; Rillig et al., 2019).

Firstly, our investigation of forests in the Western Amazonia suggests that litter prokaryotes apparently do not have an intrinsic relationship with the root layer and soil microbiota; therefore, they are not directly influenced by soil attributes. It is noteworthy that litter microbiomes are likely predominantly endophytic and related to the forests’ floristic composition and phenology patterns (Buscardo et al., 2018). A specific litter quality chemically related to the forest phytophysiognomy is added to the forest floor, providing different drivers for microbial community structuring (Buscardo et al., 2018; Ritter et al., 2018, 2020). Nonetheless, plant diversity and community composition are influenced by geology and physicochemical soil properties (Higgins et al., 2011; Ritter et al., 2018), which is indirectly important to explain variations in composition and structure of the litter microbiota. *Proteobacteria, Actinobacteria*, and *Bacteroidetes* were the most abundant phyla in that compartment, as already evidenced by Purahong et al. (2016) and Tláskal et al. (2016). Moreover, we observed differences in the communities between study sites, explained by the litter’s chemical composition. We detected a higher content of polyphenols, tannins, and C:N ratio, mainly in the litter of the MAN’s forests, which may be related to the highest relative abundance of *Actinobacteria* (Lewin et al., 2016) and a smaller abundance of *Bacteroidetes* (Xue et al., 2016) compared to the other study sites.

The fine-root production and turnover have a significant plant detritus input to the soil. It is also a key energy source to soil microbiomes, and consequently, a major pathway of nutrient flux in terrestrial ecosystems (Yuan and Chen, 2010; Zechmeister-Boltenstern et al., 2015). In our study, the root layer-associated communities also showed significant structural differences among the study sites but sharing similarities with the bulk soil due to its transient position on the forest floor. Despite the differences in community structure, we observed a clear enrichment of *Planctomycetes* in the root layer for all study sites. Some planctomycetes may be involved in degrading polymeric organic matter (Ivanova et al., 2018). However, experimental data remain scarce due to the low number of characterized representatives of this phylum. The higher relative abundance of *Planctomycetes* in the root layer has already been reported for the Amazonian rainforest (Fonseca et al., 2018). Nevertheless, more studies are needed to understand the ecological role of planctomycetes in the root layer of tropical rainforests and its potential representativity for that ecological niche.

#### Forest Floor as an Ecosystem for Accessing Microbial Diversity in Tropical Forests

Although our results agreed with previous studies that have identified higher alpha diversity in pasture soils compared to forests, a better understanding of microbial turnover and gamma “regional” diversity is still on demand, as pointed out by Petersen et al. (2019) in a recent meta-analysis that tackled the soil microbiota in tropical land uses. Our diversity partitioning analysis does not indirectly indicate a positive correlation between plant and soil prokaryotic beta diversity, as found by Prober et al. (2015), neither does it indicate the reduction of spatial heterogeneity in pastures introduced after deforestation, as evidenced by Rodrigues et al. (2013) in the Western Amazonia and Goss-Souza et al. (2017) in the Atlantic rainforest. Our results agreed with the findings described by de Carvalho et al. (2016), who found a higher beta diversity for soil prokaryotes in more altered land uses of the Eastern Amazonia, such as pastures, especially for ASV richness (q = 0) and Shannon diversity (q = 1).

Nevertheless, when forest litter and root layer were taken into account with the bulk soil, we detected a higher effective number of communities (beta diversity) within all studied forests rather than pastures. Since similar trends were found among the study regions, geographically distant and dissimilar in the composition of the measured soil and litter variables, the forest floor’s biodiversity might confer similar ecological functioning abilities to the forest ecosystem, such as nutrient cycling and C sequestration, leading to a positive diversity–stability relationship at the landscape scale. Moreover, aspects related to the forest floor’s functional redundancy are crucial for further investigation. BUJ was the only site where the global (Hill’s 0, 1, and 2) gamma diversity significantly differed between the forest floor and pasture bulk soil. The non-overlapping of the gamma diversity in less sensitive q values (q = 1 and q = 2; see Figure 6) may indicate that the higher natural fertility found in BUJ soils, in addition to the higher labile N content in its forest litter, should support a more stable prokaryotic diversity than the other study regions that only showed a significant effective number of species in the most sensitive Hill number (i.e., ASV richness, q = 0). Our results partially corroborate our second hypothesis since we have not seen consistent increases in alpha “local” and gamma “regional” diversities after including all forest floor compartments in the diversity partition analysis. Intriguingly, when observed individually, the litter, root layer, and bulk soil compartments do not give clear information about the turnover of prokaryotic communities, so an integrated interpretation of this system is necessary. Similar findings were reported by Ritter et al. (2018), where the correlation between OTU diversity in litter and soil was weak for prokaryotes and non-significant for eukaryotes. Considering the fungal communities, which play a pivotal role in tropical biodiversity (Ritter et al., 2020), Berkelmann et al. (2020) reported a decrease in the diversity of functional genes but an increase in taxonomic diversity, comparing a gradient from rainforest to agriculturally managed systems in Sumatra (Indonesia), indicating prevalence in less versatile species in monoculture soils or more functionally redundant taxa. Since the habitat type strongly shape the fungal community composition (Ritter et al., 2018, 2020), broad efforts should be made to measure a wider portion of soil biodiversity, aiming for a better understanding of the effects of land-use intensification on complex edaphic microbiomes to predict risks to the ecosystem functioning, which are essential for the maintenance of life.

## Conclusion

Altogether, our results support previous studies that show a strong relationship between soil pH and fertility on the structure of prokaryotic metacommunity in the Amazon region. This relationship was observed at the local level, as a consequence of forest-to-pasture conversion, and at the regional level, due to natural differences in soil fertility. All pasture bulk soils have prokaryotes more correlated with increases in soil pH and base saturation, resulting in higher alpha, beta, and gamma diversities. Beta and gamma diversities were generally higher in the forests when the forest floor was considered a whole, highlighting increases in microbial heterogeneity across space; however, at the plot-scale (alpha diversity), it remained higher in pasture bulk soils. By adding the forest litter and root layer to the bulk soil in our measurements, we demonstrate that prokaryotes vary in their community structure and composition among the forest floor compartments, with a relevant site-specific influence. Our findings shed light on the importance of including the forest floor compartments to understand the dynamics of microbial communities across tropical ecosystems, besides giving new perspectives on the issue of biotic homogenization. Other pasture floor compartments should be characterized and included to generate a better picture of the presented scenario for future efforts.

## Data Availability Statement

The datasets presented in this study can be found in online repositories. The names of the repository/repositories and accession number(s) can be found below: http://www.mg-rast.org/linkin.cgi?project=mgp94905.

## Author Contributions

ID, AO, JC, and EJ designed the study. AO, AB, and EJ conducted the sampling. FR, TR, SS, MC, and MF conducted the laboratory analyses. Soil descriptions and characterization were performed with support from AO, JL, PM, and WT. FR conducted the data analysis with support from AH. FR, AH, and EJ led the manuscript writing. All co-authors contributed to the drafts and gave final approval for publication.

## Conflict of Interest

The authors declare that the research was conducted in the absence of any commercial or financial relationships that could be construed as a potential conflict of interest.

## Footnotes

1 https://github.com/benjjneb/dada2
